# Ideals of the Republic

**Published:** 1889-07

**Authors:** 


**Ideals of the Republic; or, Great Words from Great Americans.** From the Knickerbocker Press of G. P. Putnam's Sons. New York. 1889.

This dainty little volume, artistically bound in blue and gold, is one of the Knickerbocker Nuggets series, and is indeed a "diminutive mass of precious metal." The younger generations are not familiar enough with the Declaration of Independence and the Constitution of the United States, and this brings them before the public in a most readable condition. The book also contains Washington's and Lincoln's inaugural addresses. The latter's second inaugural certainly deserves to be enrolled among the classics of American literature. It was an immortal utterance, worthy of the great Lincoln himself The value of this volume is enhanced by portraits of Washington and Lincoln.

